# Crystal structure of the fibre head domain of bovine adenovirus 4, a ruminant atadenovirus

**DOI:** 10.1186/s12985-015-0309-1

**Published:** 2015-05-22

**Authors:** Thanh H. Nguyen, Márton Z. Vidovszky, Mónika Z. Ballmann, Marta Sanz-Gaitero, Abhimanyu K. Singh, Balázs Harrach, Mária Benkő, Mark J. van Raaij

**Affiliations:** Departamento de Estructura de Macromoleculas, Centro Nacional de Biotecnologia (CNB-CSIC), calle Darwin 3, 28049 Madrid, Spain; Institute for Veterinary Medical Research, Centre for Agricultural Research, Hungarian Academy of Sciences, Budapest, Hungary; Department of Biological Sciences, Cork Institute of Technology, Bishopstown, Cork, Ireland; Current address: School of Biosciences, Stacey Building, University of Kent, Canterbury, Kent, CT2 7NJ United Kingdom

**Keywords:** Anomalous dispersion, Atadenovirus, Beta-sandwich, Crystallography, Fibre protein, Host-cell recognition, Isomorphous replacement, Ruminants

## Abstract

**Background:**

In adenoviruses, primary host cell recognition is generally performed by the head domains of their homo-trimeric fibre proteins. This first interaction is reversible. A secondary, irreversible interaction subsequently takes place *via* other adenovirus capsid proteins and leads to a productive infection. Although many fibre head structures are known for human mastadenoviruses, not many animal adenovirus fibre head structures have been determined, especially not from those belonging to adenovirus genera other than *Mastadenovirus*.

**Methods:**

We constructed an expression vector for the fibre head domain from a ruminant atadenovirus, bovine adenovirus 4 (BAdV-4), consisting of amino acids 414–535, expressed the protein in *Escherichia coli*, purified it by metal affinity and cation exchange chromatography and crystallized it. The structure was solved using single isomorphous replacement plus anomalous dispersion of a mercury derivative and refined against native data that extended to 1.2 Å resolution.

**Results:**

Like in other adenoviruses, the BAdV-4 fibre head monomer contains a beta-sandwich consisting of ABCJ and GHID sheets. The topology is identical to the fibre head of the other studied atadenovirus, snake adenovirus 1 (SnAdV-1), including the alpha-helix in the DG-loop, despite of them having a sequence identity of only 15 %. There are also differences which may have implications for ligand binding. Beta-strands G and H are longer and differences in several surface-loops and surface charge are observed.

**Conclusions:**

Chimeric adenovirus fibres have been used to retarget adenovirus-based anti-cancer and gene therapy vectors. Ovine adenovirus 7 (OAdV-7), another ruminant atadenovirus, is intensively tested as a basis for such a vector. Here, we present the high-resolution atomic structure of the BAdV-4 fibre head domain, the second atadenovirus fibre head structure known and the first of an atadenovirus that infects a mammalian host. Future research should focus on the receptor-binding properties of these fibre head domains.

## Background

**Fig. 1:**
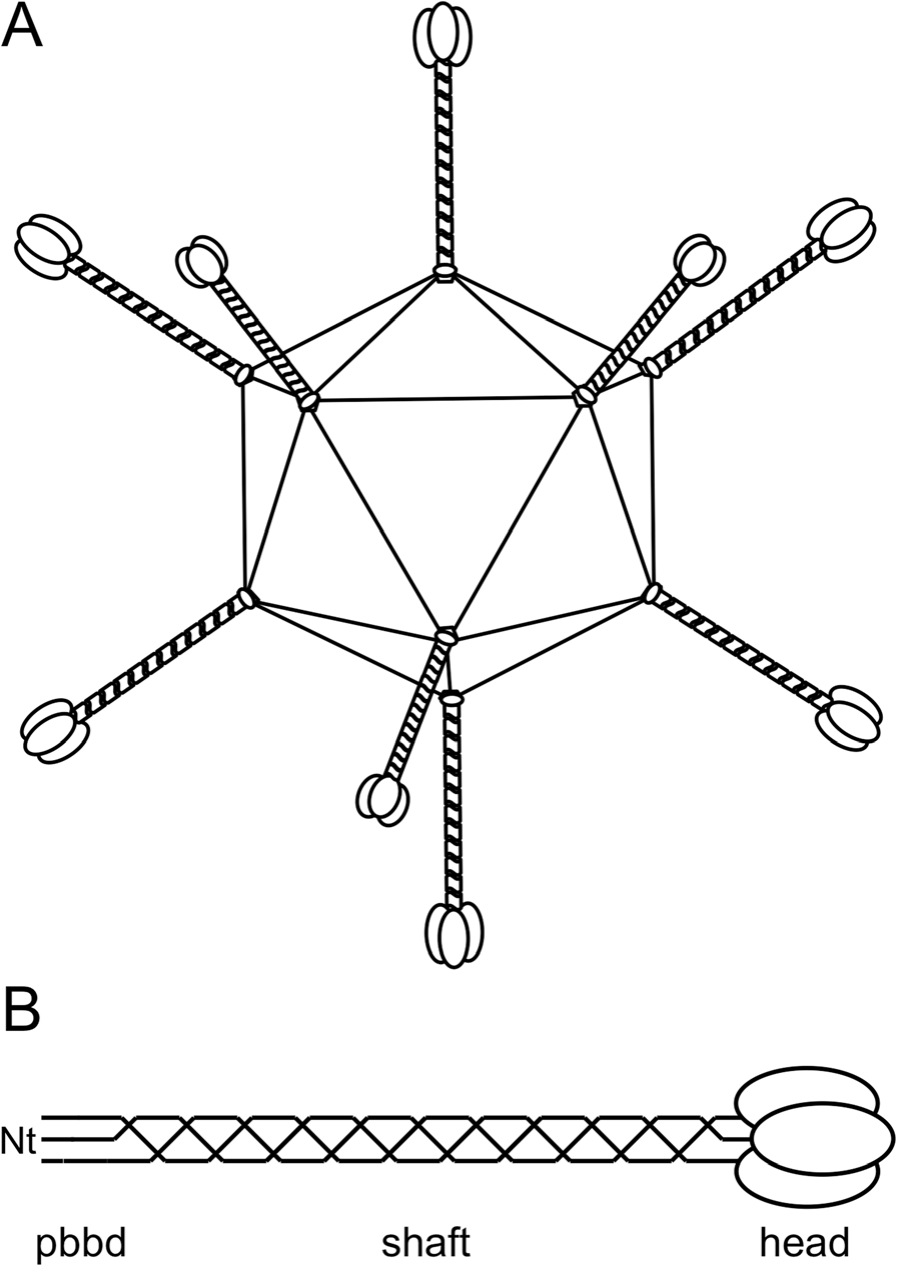
Schematic drawing of the icosahedral adenovirus (**a**) and the trimeric adenovirus fibre (**b**). The N-terminus is indicated. For BAdV-4, the penton-base binding domain (pbbd) is predicted to contain residues 1–80, the shaft domain residues 81–413, while the fibre head contains residues 420–535. A short linker (residues 414–419) probably joins the head and shaft domain

Adenoviruses have been isolated from many different vertebrate species [1–4] and, depending on the type, have been associated with respiratory, ocular and gastrointestinal infections [5, 6]. They are icosahedral non-enveloped viruses with a linear double-stranded DNA genome [7]. The twenty facets of the capsid are formed by the hexon protein (twelve trimers per facet), the vertices by a pentameric penton base complex each (Fig. 1a). From each vertex, one or more trimeric fibre proteins protrude [8]. Host cell recognition by human adenoviruses (which are all mastadenoviruses) has been widely studied [9]. Cell recognition and attachment are usually initiated by the fibre protein, via its C-terminal head domain. This attachment triggers a variety of cell responses leading to the activation of cell internalization via endocytosis [10]. When adenoviruses infect a host cell, they introduce their DNA into the host, but do not incorporate it into the host genome. This makes adenovirus a good candidate for gene therapy and other therapeutic treatments and adenovirus is widely studied and used as such [11–18]. Although fibre head structures have been determined for many different human adenovirus fibre heads [7], for animal adenoviruses only the canine adenovirus 2 [19], fowl adenovirus 1 long and short fibre heads [20, 21], the porcine adenovirus 4 fibre head and galectin domain structures [22] and the snake adenovirus 1 (SnAdV-1) fibre head [23] structures have been published, while well-diffracting crystals of the turkey adenovirus 3 fibre head have been obtained [24].

*Atadenovirus* is one of the five genera of the family *Adenoviridae* [1, 2, 25]. They are serologically and phylogenetically distinct from the other adenovirus genera, and their genomic organization also differs [1, 8, 14, 26]. Their capsids contain an extra protein (LH3), important for capsid stability [27]. Four trimeric LH3 knobs are present on each facet, with the LH3 protein being wedged in-between three hexon trimers. Atadenoviruses have been detected in a broad range of hosts, including predominantly scaled reptiles (order Squamata), as well as birds, ruminants and a marsupial [16, 26, 28–35]. So far, five species of atadenovirus have been confirmed: *Snake atadenovirus A*, *Duck atadenovirus A, Bovine atadenovirus D, Ovine atadenovirus D* and *Possum atadenovirus A* [2, 3, 36].

About half of identified ruminant adenoviruses are mastadenoviruses, while the remainder are atadenoviruses [2]. From the bovine adenoviruses, serotypes 1, 2, 3, 9 and 10 are mastadenoviruses [37, 38], while bovine adenoviruses 4, 5, 6, 7 and 8 are atadenoviruses [39, 40]. Bovine adenovirus 4 (BAdV-4; strain THT/62) is the reference strain for the *Bovine atadenovirus D* species and was first isolated and characterized in Hungary (GenBank accession number AF036092) [39, 41, 42]. BAdV-4 contains a single fibre gene, encoding a protein of 535 amino acids in length with low sequence identity to known adenovirus fibres (15–19 %). Like other adenovirus fibres, it is predicted to consist of three domains: an N-terminal penton-base binding domain, a shaft domain and a C-terminal fibre head domain (Fig. 1b). Based on the location of the triple beta-spiral shaft repeats [43, 44], the N-terminal penton-base-binding domain of the fibre is proposed to contain amino acids 1–80, while the C-terminal fibre head domain was expected to start around residue 414. Here, we report the expression, purification, crystallization and structure solution of the fibre head domain of BAdV-4 at 1.2 Å (0.12 nm) resolution. It is the second atadenovirus fibre head structure that has been solved, after that of the SnAdV-1 fibre head domain [23]; and the first of an atadenovirus which infects mammalian cells. Although the secondary structure topology is the same as for other adenovirus fibre heads, differences are observed in the loops connecting the beta-strands and in the predicted surface charge. Our structure is a first step towards a better understanding of BAdV-4 host cell interaction, which, in turn, will have implications for the use of mammalian atadenovirus for medical purposes, be it in the use of whole viruses [14], or in chimeric fibres with atadenovirus fibre heads [45].

## Results and discussion

### Purification, crystallization and structure solution of the BAdV-4 fibre head

**Fig. 2:**
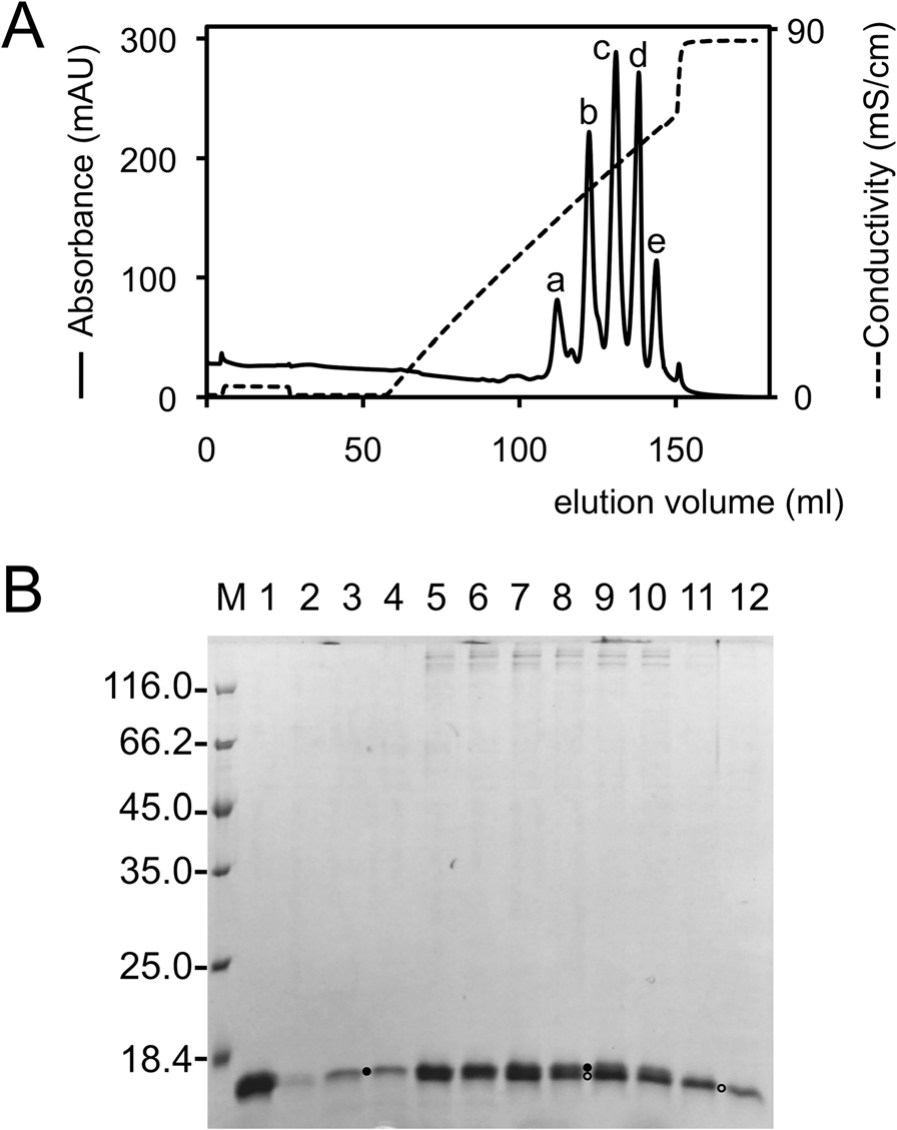
Purification of BAdV-4 fibre head protein by cation exchange chromatography. **a**. Elution profile. The absorbance measured at 280 nm and the conductivity are shown. **b**. Denaturing gel electrophoresis of peak fractions. In lane M, molecular weight markers of the size (in kDa) indicated on the left were loaded. In lane 1, an aliquot of the sample after nickel-affinity chromatography was loaded, eluted with imidazole. In lane 2, an aliquot of the sample eluted from the same column at pH 5 was loaded. In lanes 3 and 4, lanes 5 and 6, lanes 7 and 8, lanes 9 and 10 and lanes 11 and 12 samples from peaks a, b, c, d and e were loaded, respectively. Samples in odd lanes were heated for 5 min at 95 °C before loading, samples in even lanes were not. Putative upper and lower bands are indicated with filled and open circles, respectively. The protein runs as a monomer in all cases: the BAdV-4 fibre head does not form denaturant-stable trimers like other adenovirus fibre heads

Sequence analysis suggested that residues 414–535 might form the C-terminal head domain. Therefore, expression vectors for the full-length sequence (1–535), a C-terminal fragment with part of the predicted shaft domain (residues 248–535) and the putative fibre head domain alone (amino acids 414–535) were constructed. All three constructs expressed the expected protein, but soluble protein was only obtained for the putative fibre head domain. Expression was carried out at low temperature and the protein was purified by metal affinity chromatography and ion exchange chromatography as described in the Methods section. In cation exchange chromatography, the protein eluted in five different peaks (Fig. 2). N-terminal sequence analysis by Edman degradation of protein from the five peaks yielded the same sequence (GSSHH), suggesting the N-terminal six-histidine tags to be intact and the N-terminal Met to be removed upon expression in *E. coli*. Mass spectrometry on protein from the five peaks suggested adducts of around 180 Dalton, which could correspond to spontaneous alpha-*N*-6-phosphogluconoylation of the six-histidine tag, as observed for other proteins with the same N-terminal six-histidine tag [46]. Multiple peaks eluting from cation chromatography were also observed for a different protein expressed in *E. coli* with the same N-terminal six-histidine tag [46]. Careful inspection of the gel in Fig. 2b suggests the presence of two bands. The presence of two bands may well be caused by differential alpha-*N*-6-phosphogluconoylation. The fact that the protein is a trimer may lead to mixed species and thus the generation of the multiple peaks observed. The fact that the earlier peaks contain more adduct is consistent with the cation exchange chromatography behaviour, because the adduct would be expected to remove a positive charge and lead the protein to elute at lower salt concentrations. Protein from the three major peaks b, c and d was concentrated separately to 12 mg/ml (peaks a and e were considered to contain too little protein for successful crystallization). Around 13 mg of purified protein in total was obtained per litre of expression culture. Adenovirus fibre heads eluting in several peaks from ion exchange chromatography has been observed before [24, 47].

Well-diffracting crystals were obtained from all of the three major cation exchange elution peaks at 21 °C, by sitting drop vapour diffusion from precipitant solutions containing 20 % poly-ethylene glycol 3350 and either 0.2 M potassium thiocyanate or 0.2 M sodium isothiocyanate. Crystals appeared after three days and grew to their maximal size in about three weeks. They were found to belong to space group *P*1, with one protein trimer in the unit cell, leading to a solvent content of 44 % and a Matthews coefficient of 2.2 [48]. A calculated self-rotation function confirmed the presence of non-crystallographic three-fold symmetry in the crystal. We were not successful in solving the structure by molecular replacement, although the availability of a high-quality isomorphous derivative dataset meant we did not pursue this method extensively. High-resolution isomorphous datasets were collected from the native and derivative crystals, and the derivative dataset contained high-quality anomalous signal, which allowed automated structure solution by single isomorphous replacement with anomalous dispersion. The final refined model contains residues 420–534 from each of the three protein chains in the trimer, plus ordered solvent atoms. No reliable density was observed for the N-terminal purification tag, for residues 414–419 or for the C-terminal glutamine (amino acid 535), suggesting that these are disordered. No differences were observed between crystallization success, crystal shape or form, for protein obtained from the 3 central peaks; which is consistent with that they presumably only differ in the adduct on the disordered N-terminal purification tag, for which there is room in the crystal lattice. The non-observed residues 414–419 may form a linker between the shaft and head domain in the intact fibre protein. Data collection, phasing and refinement statistics are shown in Table 1.

**Table 1:** Crystallographic data collection, phase determination, solvent flattening and refinement statistics (all values in parenthesis are for the highest resolution bin)

Data collection	Native	Derivative
Cell parameters (a, b, c) (Å)	43.3, 48.2, 52.1	43.4, 48.4, 52.6
Cell parameters (α, β, γ) (°)	117.1, 95.6, 110.2	116.9, 95.8, 110.2
Wavelength (Å)	0.9763	1.0023
Resolution (Å)	44.2–1.17 (1.21–1.17)	26.2–1.40 (1.42–1.40)
Observed reflections	103174 (9613)	62174 (2058)
Multiplicity	3.6 (3.5)	3.4 (3.2)
Completeness (%)	91.2 (86.7)	92.6 (61.0)
Rmerge (%)	3.6 (43.1)	4.8 (22.4)
<I/sigma(I)>	15.7 (2.6)	12.5 (3.8)
Wilson B (Å^2^)	10.5	12.0
CC1/2	1.000 (0.842)	0.995 (0.894)
CCanom	−0.078 (0.008)	0.442 (0.131)
Phase determination		
Number of heavy atom sites		6 Hg
Phasing power (isomorphous/anomalous)		2.290/1.716
Figure of merit		0.601
Solvent flattening (40.8 % solvent)		
Hand score (original/inverted)		0.3647/0.8403
Overall correlation on |E|^2^/contrast		2.6012
Refinement		
Resolution range (Å)	44.2–1.17 (1.22–1.17)	
No. reflections used in refinement	100391 (11648)	
No. reflections used for R-free	2782 (311)	
R-factor (%)	11.6 (21.7)	
R-free (%)	14.6 (23.9)	
No. of protein/solvent atoms	2841/448	
Average B protein/solvent atoms (Å^2^)	18.7/35.5	
Ramachandran plot (favoured/allowed) (%)	98.0/99.7	
R.m.s. deviation of bonds (Å) and angles (°)	0.012/1.5	
PDB code	4UE0	

### Overall structure

**Fig. 3:**
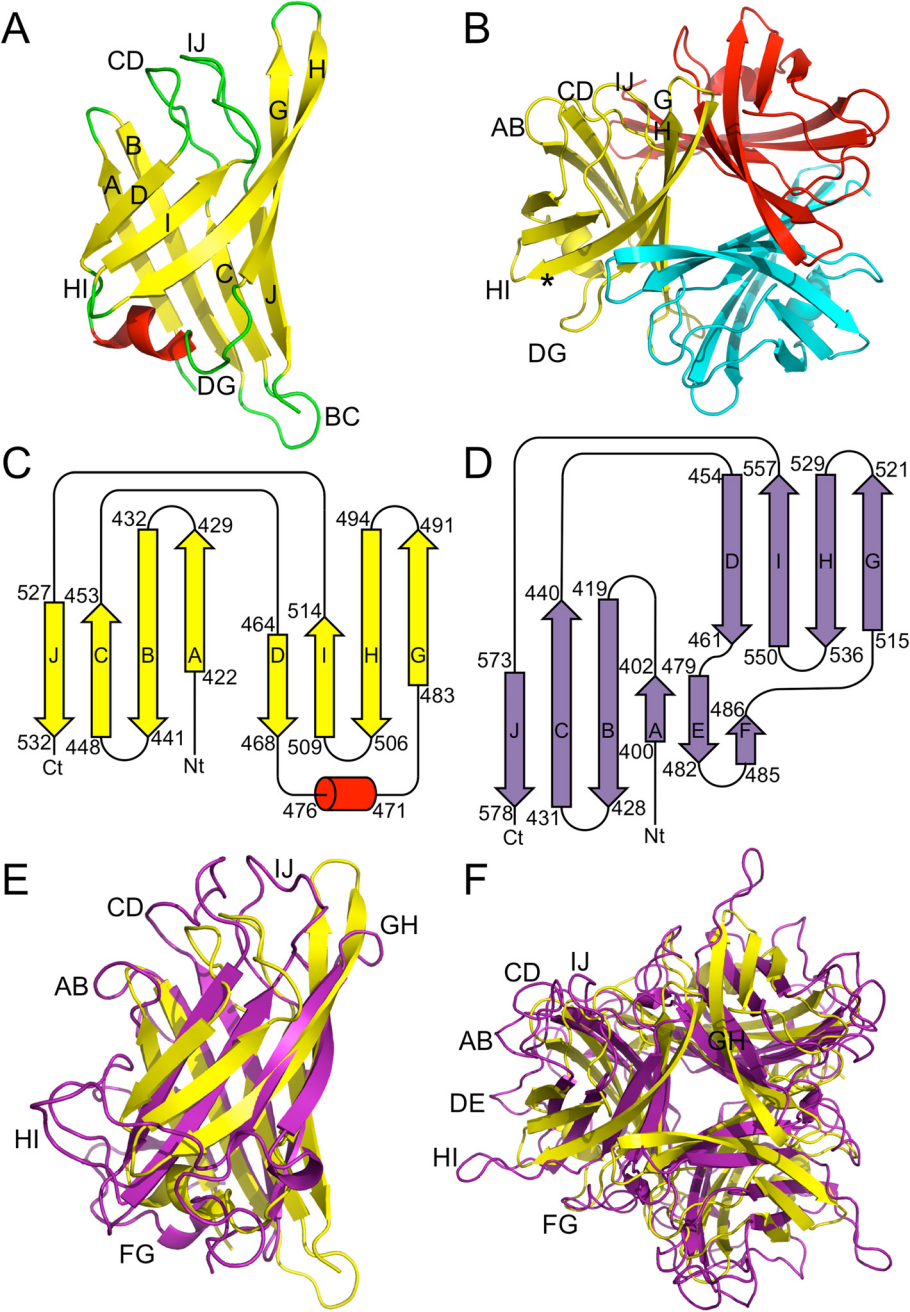
Structure of the BAdV-4 fibre head domain. **a**. Monomer structure coloured by secondary structure. Beta-strands and most of the loops are labelled. **b**. Top view of the trimer with the three monomers coloured differently. An asterisk indicates the GHID-sheet in the yellow monomer and most loops are labelled. **c**. Topology diagram of the BAdV-4 fibre head, with start and end residues of each beta-strand and of the alpha-helix labelled. **d**. Topology diagram of the HAdV-5 fibre head, shown for comparison. **e**. Superposition of the HAdV-5 fibre head monomer onto the BAdV-4 fibre head monomer in the same orientation as part A. **f**: Superposition of the HAdV-5 fibre head trimer onto the BAdV-4 fibre head trimer (top view). Surface loops of the HAdV-5 fibre head trimer are indicated

**Fig. 4:**
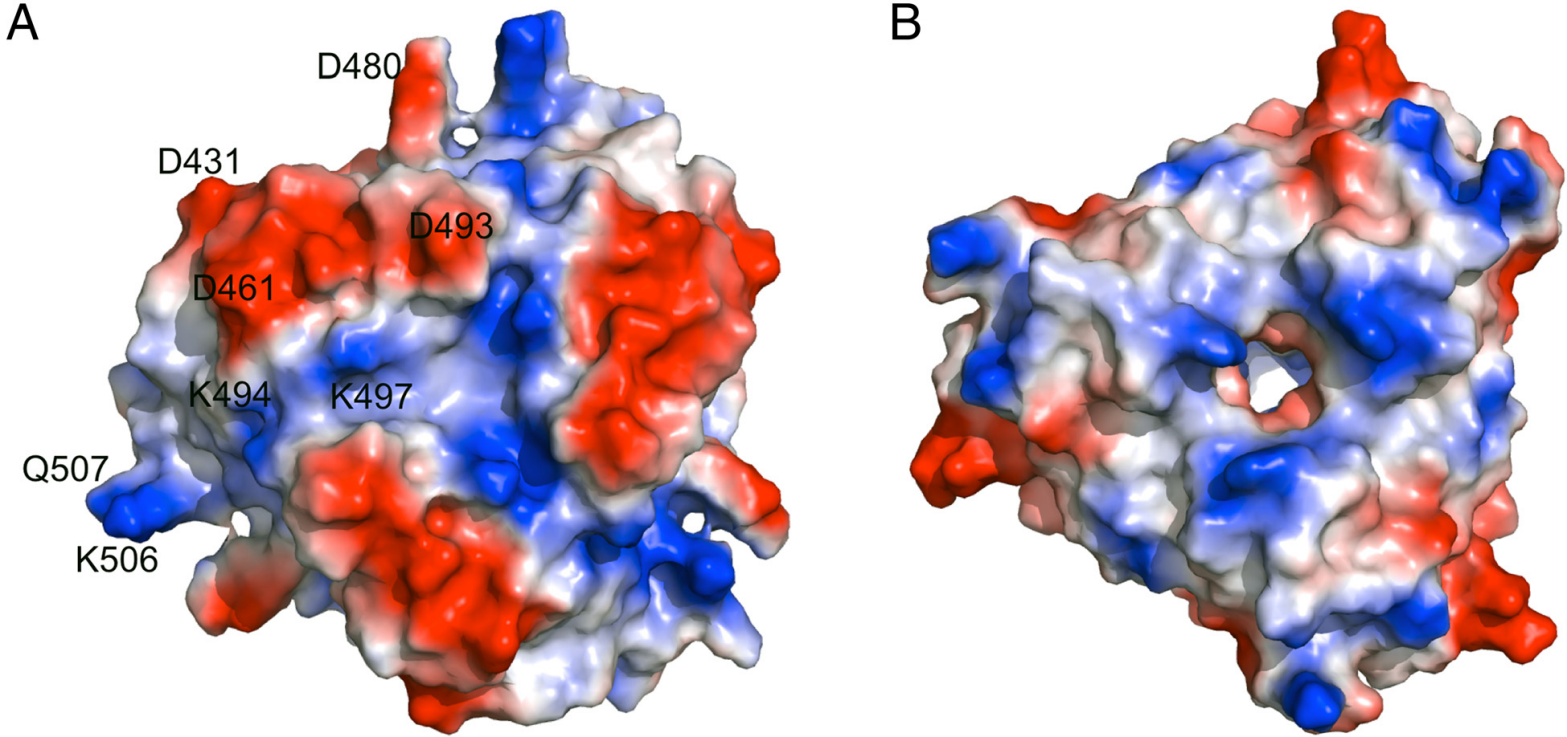
Qualitative electrostatic surface of the BAdV-4 fibre head trimer (**a**) and SnAdV-1 fibre head trimer (**b**) seen from the top. Some BAdV-4 fibre head surface residues that are expected to have negative or positive charges are labelled

The structure of the BAdV-4 fibre head domain is composed of three monomers, associated into a compact trimer (Fig. 3). Each monomer consists of a beta-sandwich of two beta-sheets (ABCJ- and GHID-sheets; Fig. 3a), in the same topology (Fig. 3c) as other adenovirus fibre heads (the topology of the HAdV-5 fibre head is shown as an example in Fig. 3d) [7, 47, 49]. The same topology is also shared with reovirus fibre heads [50, 51], the bacteriophage PRD1 fibre head [52] and lactobacillus phage receptor-binding domains [53, 54], as previously described [23]. The loop between strands D and G is longer than the others and contains an alpha helix (residues 471–476), instead of the E- and F-strands that many mastadenovirus fibre head domains have in this loop (Fig. 3d) [49]. The A-, B-, G- and H-strands are relatively long (eight, ten, nine and 13 residues, respectively), while the C-, D-, I- and J-strands are shorter (six, five, six and six residues, respectively). The AB-, BC-, GH- and HI-loops are short beta-turns of four amino acids each, while the CD-, DG- and IJ-loops are longer (twelve, 16 and 14 residues, respectively). Similar as observed for other adenovirus fibre heads, the CD- and IJ-loops are located at the top of the fibre head, whereas the DG-loop is located on the side. All are potentially involved in receptor interactions. The CD- and IJ-loops run parallel to each other and contain negatively charged residues, forming electronegative patches on the protein surface (Fig. 4a). The CD- and IJ-loops are also involved in inter-monomer contacts with the J-strand of the neighbouring monomer.

The two beta-sheets of the BAdV-4 fibre head monomer face each other at an angle of around 120°. The ABCJ-sheet is mostly on the “inside”, and only a part of the AB beta-hairpin is solvent-accessible. The rest of the AB beta-hairpin and the whole C- and J-strands are buried and extensively involved in inter-monomer contacts. In contrast, most of the GHID-sheet is solvent-accessible (Fig. 3b). Together with the alpha-helix, the two beta-sheets from each monomer form a compact globular trimeric beta-propellor.

### Comparison with other adenovirus fibre heads

Mastadenovirus fibre heads are larger than atadenovirus fibre heads, as previously described [23], mainly due to the surface loops being longer. The AB-, CD-, DG-, HI- and IJ-loops are all shorter in the BAdV-4 fibre head than in the prototype mastadenovirus fibre head structure, HAdV-5. Exceptions are strands G and H and the BC-loop, which are longer in the BAdV-4 fibre head. The low similarity of the structures makes it impossible to make meaningful speculations about receptor binding.

**Fig. 5:**
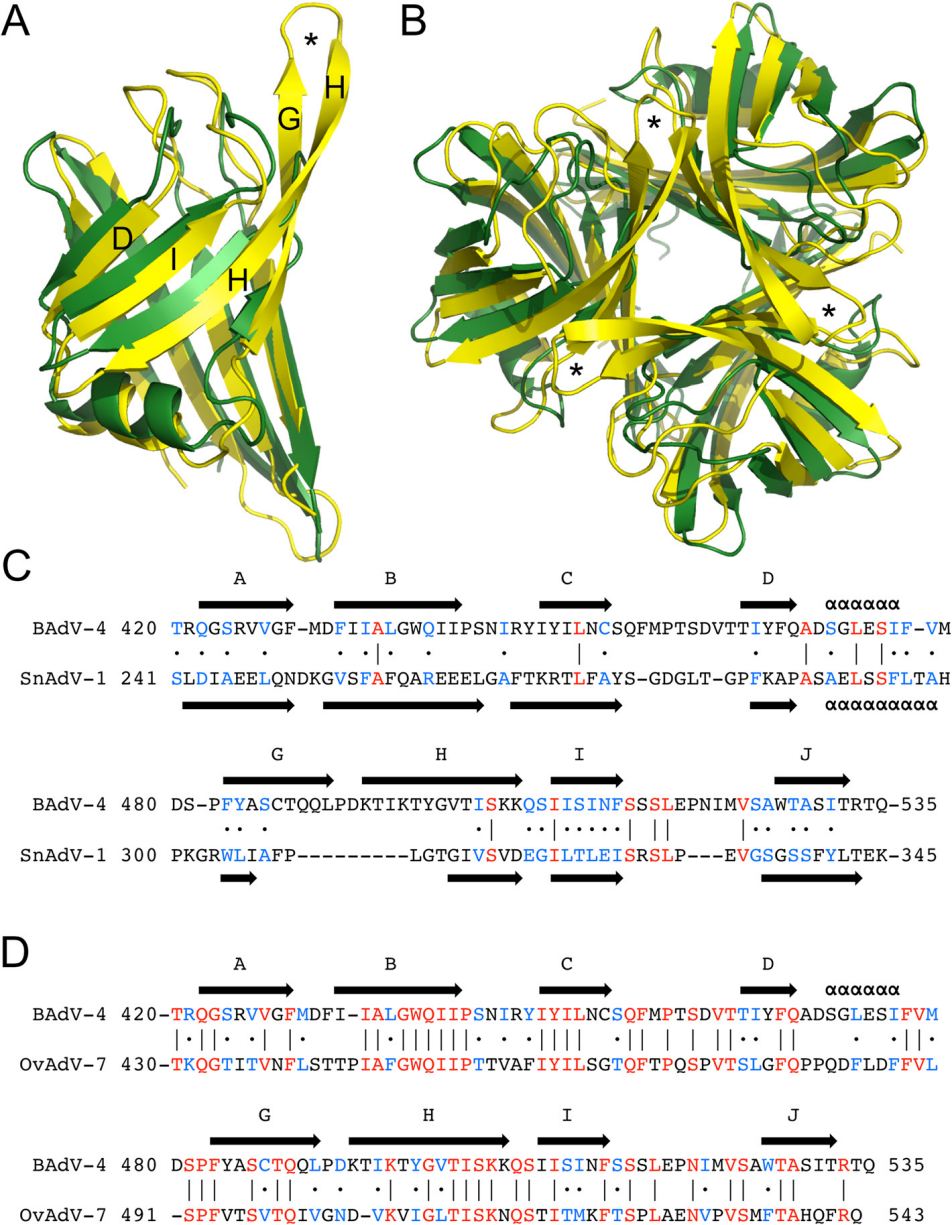
Comparison of the BAdV-4 fibre head with other atadenovirus fibre heads. **a**. Superposition of the BAdV-4 monomer onto the SnAdV-1 monomer (same orientation as in Fig. 3**a**). The BAdV-4 fibre head is shown in yellow and the SnAdV-1 fibre head in dark-green. The strands of the GHID-sheet are labelled. **b**. Superposition of the BAdV-4 trimer onto the SnAdV-1 trimer (same orientation as in Figs. 3**b** and **f**, top view). The colours are as in panel A. In panels A and B, asterisks indicate the location of the GH-loops. **c**. Structure-based sequence alignment of the BAdV-4 and SnAdV-1 fibre head sequences. Strands are indicated with arrows and labelled; alpha-helices are indicated with α’s. Similar residues are coloured blue and indicated with the symbol · (a dot); identical residues coloured red and marked with the symbol | (a line). **d**. Sequence alignment of the BAdV-4 and OAdV-7 fibre head sequences

The BAdV-4 fibre head shares only 15 % sequence identity with the other atadenovirus fibre head (from SnAdV-1) for which a structure is known [23]. Nevertheless, the structure is very similar (Fig. 5). This is surprising considering that today one of these viruses exists in cattle (after a supposed host switch from reptiles) [31, 55] and the other still in snakes, with which they presumably continuously co-evolved. The two trimers can be superposed with an overall Z-score of 13 and a root mean square deviation (r.m.s.d.) of around 2 Å. When monomers are superposed, the r.m.s.d. is only slightly lower (1.8 Å), indicating that the relative orientation of the monomers in the trimer is also very similar. The topologies of both fibre heads are identical, with a conserved beta-sandwich motif and an alpha-helix in the DG-loop. Both fibre heads share the same 120° angle between two beta-sheets, but differences are observed in the length and the conformation of the loops.

The CD- and IJ-loops of the BAdV-4 fibre head are longer than those in the SnAdV-1 fibre head. In contrast, the DG-loop of the BAdV-4 fibre head is two amino acids shorter than its SnAdV-1 counterpart. A noticeable difference is observed in the length of the G- and H-strands, which are quite a bit longer in the BAdV-4 fibre head (9 vs. 5 for the G-strand and 13 vs. 8 residues for the H-strand). As mentioned before, the CD-, IJ- and GH-loops are all located on the top of the molecule, while the DG-loop is on the side, making all of them potentially important for receptor interaction. In a structure-based alignment (Fig. 5c), the lack of sequence similarity becomes even more evident, except for the very C-terminal part of the protein (end of the H-strand and the I- and J-strands and the HI-turn). It may be speculated that this conserved part of the protein is important for initiation of protein folding and has therefore diverged less. When calculated electrostatic surfaces are considered, the BAdV-4 fibre head has distinct electro-negatively charged patches on its surface (Fig. 4a), while the SnAdV-1 surface is more electro-positive (Fig. 4b) [23]. This may have implications for receptor-binding and suggest they do not bind the same receptor, although the nature of the receptors for neither of the viruses is known.

From previous observations in other adenoviruses, the BAdV-4 fibre head was expected to be a stable protein. However, unlike other head domains such as that of the fowl adenovirus 1 long fibre [56] and of the SnAdV-1 fibre [57], this trimer is not denaturant-stable at room temperature (Fig. 2b). Its melting temperature, as measured by a thermofluor assay, was estimated to be 67 °C, which is relatively high. In the same assay, no unfolding transition was observed for the SnAdV-1 fibre head below 95 °C, confirming the SnAdV-1 fibre head is more stable. Each monomer has a total solvent accessible surface area of 6.6 × 10^3^ Å^2^, out of which 0.62 × 10^3^ Å^2^ (9 %) gets buried upon trimer formation. In the SnAdV-1 fibre head, about 1.5 times more surface area is buried [23]. No salt bridges are formed upon trimeric assembly for the BAdV-4 fibre head, while in the SnAdV-1 fibre head a strong bidentate salt bridge between Arg304 and Glu333 of neighbouring monomers is present, which may explain why the SnAdV-1 fibre head is more stable than the BAdV-4 fibre head. Table 2 lists the identified interactions, from which it can be concluded that the SnAdV-1 fibre head has more hydrophobic, aromatic and ionic interactions between monomers, while the number of inter-monomer hydrogen bonds is the same.

**Table 2:** Potential inter-monomer interactions of atadenovirus fibre heads

	SnAdV-1 fibre head	BAdV-4 fibre head
Hydrophobic interactions (<5 Å)	Leu265 - Ala245	Ile445 - Val246
	Phe268 - Ala245	Tyr484 - Ile496
	Phe268 - Ala257	Tyr484 - Met523
	Pro301 - Tyr276	
	Leu306 - Pro310	
	Leu306 - Leu311	
	Ala308 - Pro310	
	Phe340 - Phe274	
	Phe340 - Tyr276	
	Tyr341 - Tyr276	
	Leu342 - Ala257	
	Leu342 - Phe274	
	Leu342 - Tyr276	
Hydrogen bonds	Lys239NZ - Ser241O	Arg446NH2 - Leu436O
	Lys239NZ - Asp243OD2	Pro482O - Gln482NE2
	Lys270NZ - Gln259OE1	Tyr484N - Gln455OE1
	Lys302NZ - Gly278O	Tyr484OH - Gln490N
	Arg304NH1 - Glu333OE1	Tyr484OH - Thr488OG1
	Arg304NH1 - Glu333OE2	Ser530O - Gln455N
	Arg304NH2 - Glu333OE1	Ser530OG - Ser454OG
	Phe340N - Ser336OG	Thr532OG1 - Ser454OG
Ionic interactions (<6 Å)	Lys239 - Asp243	
	Glu263 - Arg261	
	Lys302 - Asp279	
	Lys302 - Glu333	
	Arg304 - Glu333	
Aromatic interactions (4.5–7 Å)	Phe340 - Phe274	
	Phe340 - Tyr276	
	Tyr341 - Tyr276	
Cation-pi interactions (<6 Å)	Lys270 - Phe274	

The first residue is from chain A, the second from chain B of the trimer; letters behind the residue number indicate the atom, where appropriate

Ovine adenovirus 7 (OAdV-7), another ruminant atadenovirus, is intensively tested as a basis for a gene therapy vector [16], but the structure of its fibre head is unknown. Our BAdV-4 fibre head structure is likely similar to that of the OAdV-7 fibre head [27], with which it shares 44 % sequence identity (51 out of 116 amino acids; Fig. 5d). It should now be possible to build a reliable structural model of the OAdV-7 fibre head in order to identify surface residues possibly involved in receptor binding and mutating them. Our cloning, expression and crystallization strategy may also be used to obtain crystals for the OAdV-7 fibre head, and the structure determined experimentally by molecular replacement using data collected on these crystals.

## Conclusion

The high resolution structure of BAdV-4 fibre head is the second solved structure of an atadenovirus fibre head domain. However, it is the first fibre head structure of an atadenovirus which infects a mammalian host. The structure showed that the atadenovirus fibre head structure is conserved, including the alpha-helix in the DG-loops, between two species infecting very different hosts, even though the sequence identity is very low. Differences in the conformation of surface loops and in the predicted surface charge may be of importance for primary receptor recognition.

## Methods

### Cloning, expression and purification

BAdV-4 strain THT/62 [39] was propagated on primary or low-passage-number calf testis cell cultures, then the virus was purified by ultracentrifugation, and the DNA extracted as described earlier [41]. Three genome fragments, coding for the full BAdV-4 fibre protein or parts of it containing the putative head domain (UniProt Q997H2) were amplified from the extracted viral DNA (GenBank accession No AF036092) [58] by polymerase chain reaction (PCR) using three forward primers including a BamHI restriction site and a reverse primer with a HindIII restriction site. The amplified PCR products were cloned into the expression vector pET28a(+) (Novagen, Merck, Darmstadt, Germany), previously digested with the same restriction enzymes. The inserts of the resulting plasmids were sequenced and found to be correct. The pET28a(+) vector provides an N-terminal six-histidine tag.

For protein expression, *E. coli* strain JM109(DE3) was transformed with the respective expression vector and bacteria were grown aerobically at 37 °C until the optical density at 600 nm reached 0.5–0.8. At this point, the culture was cooled on ice for 30 min, IPTG was added to a final concentration of 0.5–1 mM and aerobic growth was continued for 18–20 h at 16 °C. Cells from 2 l of culture were harvested by centrifugation (10 min 5000 × *g*), re-suspended in buffer A (10 mM Tris–HCl pH 7.5, 0.5 M sodium chloride, 10 % (v/v) glycerol) including 20 mM imidazole and stored at −20 °C. After thawing, cells were lysed by two passes through a French press at about 7 MPa. Cell rests were removed by centrifugation for 30 min at 20000 × *g*.

For purification, 2 ml of nickel-NTA resin slurry (BioRad, Madrid, Spain) was added to the protein-containing supernatant and incubated with occasional gentle shaking for 30 min on ice. The resin was then transferred to a column and washed with 30 ml of buffer A with 20 mM imidazole. BAdV-4 fibre protein variants were eluted using a step-gradient of imidazole in buffer A (50 mM, 100 mM, 250 mM and 500 mM imidazole; steps of 5 ml). After analysis by denaturing gel electrophoresis, fractions containing 100–500 mM imidazole were pooled, dialysed against 20 mM MES pH 6.5 and loaded onto a Resource S6 column (GE-Healthcare Biosciences, Uppsala, Sweden). The protein was eluted with a linear gradient of 0–1 M sodium chloride in 20 mM MES pH 6.5. Fractions containing pure protein were concentrated to 18 mg/ml using an Amicon Ultra concentrator with a molecular weight cut-off of 10 kDa (Millipore, Madrid, Spain). Three washes with 10 ml 10 mM Tris–HCl pH 7.5, 50 mM sodium chloride and 5 % (v/v) glycerol were applied. The sample was stored at 4 °C prior to crystallization trials.

### Thermofluor assay

A thermal shift assay [59] was carried out in an iCycler iQ PCR Thermal Cycler (Bio-Rad, Hercules CA, USA) in the presence of the fluorescent dye SYPRO Orange (Life Technologies SA, Madrid, Spain). Reaction volumes of 30 μl were prepared in 200 μl with 30 μg of protein and 5X SYPRO Orange from the supplied 5000X stock solution. Thermal denaturation curves were obtained by heating the samples from 20 °C to 95 °C with a ramp rate of 1 °C/min and monitoring the fluorescence at every 0.5 °C increment. The melting temperature Tm is defined as the point where the slope of the fluorescence increase is maximal.

### Crystallization, crystallographic data collection and structure solution

The BAdV-4 fibre head protein was crystallized using the sitting drop vapour diffusion method (robotic setup with a Genesis RSP 150 workstation; Tecan, Männedorf, Switzerland or by manual setup). In either case, 50 μl of reservoirs were employed, and drops were prepared containing 0.2 μl of protein sample and 0.2 μl of the respective reservoir solution for robotic setups and 0.6 μl of protein plus 0.6 μl of reservoir for manual setups. Crystals were harvested with Litholoops (Molecular Dimensions, Newmarket, England) or Micromounts (Mitegen, Ithaca, New York, USA), transferred to cryo-protection solution (reservoir solution containing 20 % (v/v) glycerol) and flash-cooled in liquid nitrogen.

A heavy atom derivative was prepared by adding a few grains of methylmercury chloride to the reservoir of one drop and letting the drop equilibrate with the reservoir overnight. Two μl of reservoir solution was then added to the drop and incubated for about 5 min. The crystal was harvested in cryo-solution without methylmercury chloride as described for the native.

Crystallographic data were collected from a native and a methylmercury chloride derivative crystal at the BL13-XALOC beamline of the ALBA synchrotron [60], using a wavelength at which significant anomalous signal from the added mercury atoms was expected (1.0023 Å). Later, a higher-resolution native dataset was collected at beamline ID29 of the ESRF [61]. Crystallographic data collected at BL13-XALOC were integrated using iMosflm [62] and further processed using POINTLESS, AIMLESS and TRUNCATE [63] from the CCP4-suite [64]. Data collected at ID29 were processed using XDS [65, 66], analyzed using AIMLESS and processed by TRUNCATE to obtain structure factor amplitudes. A self-rotation function was calculated with MOLREP [67].

Structure solution was done using AUTOSHARP [68], which employs SHELX for heavy atom positions substructure determination [69], SHARP for phase determination [70] and SOLOMON for solvent flattening [71]. Reflections were selected in thin shells for calculation of Rfree [72]. Phases from AUTOSHARP were combined with the structure factor amplitudes from the high-resolution native dataset and input into ARPWARP for automated model building [73]. This obtained model was completed using COOT [74] and structure refined using REFMAC5 [75], including anisotropic temperature factor refinement. Validation was done with MOLPROBITY [76]. Structure comparisons, including r.m.s.d. and Z-score calculations, were performed using the DALI server [77]. Figures were made using PYMOL (The PYMOL Molecular Graphics System, Version 1.5.0.4. Schrödinger, LLC). Protein assembly parameters were calculated using PISA [78] and individual interactions identified with PIC [79].
